# Incidence and predictors of mortality among neonates referred to comprehensive and specialized hospitals in Amhara regional state, North Ethiopia: a prospective follow-up study

**DOI:** 10.1186/s13052-021-01139-9

**Published:** 2021-09-15

**Authors:** Alex Yeshaneh, Bizuayehu Tadele, Bogale Dessalew, Mulunesh Alemayehu, Awraris Wolde, Addisu Adane, Solomon Shitu, Haimanot Abebe, Daniel Adane

**Affiliations:** 1 Departments of Midwifery, College of Medicine and Health Sciences, Wolkite University, Wolkite, Ethiopia; 2 Departments of Public health, College of Medicine and Health Sciences, Debra Markos University, Debra Markos, Ethiopia; 3 Departments of Health Service Managment, Kotebe Metropolitan University Menelik II Medical and Health science college, Addis Ababa, Ethiopia; 4 Departments of Public Health, College of Medicine and Health Sciences, Wolkite University, Wolkite, Ethiopia

**Keywords:** Referred neonate, Transportation, Referral, Predicting factors

## Abstract

**Background:**

Neonatal mortality is a major global public health problem. Ethiopia is among seven countries that comprise 50 % of global neonatal mortality. Evidence on neonatal mortality in referred neonates is essential for intervention however, there is no enough information in the study area. Neonates who required referral frequently became unstable and were at a high risk of death. Therefore, this study aimed to assess the incidence and predictors of mortality among referred neonates.

**Method:**

A prospective follow-up study was conducted among 436 referred neonates at comprehensive specialized hospitals in the Amhara regional state, North Ethiopia 2020. All neonates admitted to the selected hospitals that fulfilled the inclusion criteria were included. Face-to-face interviews, observations, and document reviews were used to collect data using a semi-structured questionnaire and checklists. Epi-data™ version 4.2 software for data entry and STATA™ 14 version for data cleaning and analysis were used. Variables with a *p*-value < 0.25 in the bi-variable logistic regression model were selected for multivariable analysis. Multivariable analyses with a 95% confidence level were performed. Variables with *P* < 0.05 were considered statistically significant.

**Result:**

Over all incidence of death in this study was 30.6% with 95% confidence interval of (26.34–35.16) per 2 months observation. About 23 (17.83%) deaths were due to sepsis, 32 (24.80%) premature, 40 (31%) perinatal asphyxia, 3(2.33%) congenital malformation and 31(24.03%) deaths were due to other causes. Home delivery [AOR = 2.5, 95% CI (1.63–4.1)], admission weight < 1500 g [AOR =3.2, 95% CI (1.68–6.09)], travel distance ≥120 min [AOR = 3.8, 95% CI (1.65–9.14)], hypothermia [AOR = 2.7, 95% CI (1.44–5.13)], hypoglycemia [AOR = 1.8, 95% CI (1.11–3.00)], oxygen saturation < 90% [AOR = 1.9, 95% (1.34–3.53)] at admission time and neonate age ≤ 1 day at admission [AOR = 3.4, 95% CI (1.23–9.84) were predictors of neonatal death.

**Conclusion:**

The incidence of death was high in this study. The acute complications arising during the transfer of referral neonates lead to an increased risk of deterioration of the newborn’s health and outcome. Preventing and managing complications during the transportation process is recommended to increase the survival of neonates.

## Introduction

Neonatal mortality is the total number of children who died between birth and the 28th day of life. It consists of early neonatal mortality for deaths in the first week and late neonatal mortality for deaths in the following 3 weeks [1, 2]. On average 2.5 million newborns die within 28 days of life and account for 47% of the under 5 mortality in 2018. More than 99% of deaths occurred in developing countries. Sub-Saharan Africa carries more than 52% of neonatal mortality. Ethiopia is among the seven countries comprising 50% of the global neonatal mortality [3].

Global efforts that are currently underway have been declining neonatal mortality to 19 deaths per 1000 live births in 2015 to 18 deaths per 1000 live births in 2018. However, neonatal mortality remains unacceptably high in many low- and middle-income countries. The neonatal mortality rate in developing countries is more than eight times that in developed countries. In Sub-Saharan African countries neonatal death occurred in 28 deaths per 1000 live births. In Ethiopia according to the mini EDHS 2019, neonatal mortality in Ethiopia was 30 death/1000 live births [3, 4].

Worldwide, preterm birth complications, intrapartum birth complications and sepsis are the leading causes of neonatal death [5]. In Ethiopia, the top causes of neonatal death are asphyxia, complications related to prematurity and neonatal sepsis [4, 6]. Neonatal mortality related to instability or complications secondary to referral is another challenge and a worldwide health problem [7]. The severity of the problem varies from developed to developing countries [8].

The Ethiopian federal ministry of health planned to achieve neonatal mortality of < 12 deaths /1000 live births with a commitment to end preventable child death by 2030. Integrated management of neonatal and childhood illness (IMNCI), community-based nutrition (CBN), community-based newborn care (CBNC), increasing access and quality of primary health care services, early antenatal care and interventions such as referral of high-risk and sick babies to higher equipped facilities, increases in vaccination, skilled birth attendance and option B+ for PMTCT are among the strategies implemented by the government of Ethiopia. However, neonatal mortality still continues increase from 29 to 30 deaths per 1000 live births between 2016 and 2019 and varies across regions. According to research finding neonatal mortality in the Amhara regional state is 20.3% [4, 9–11].

In addition to major causes of neonatal death, some evidence in different parts of the world indicates acute physiologic complications like hypothermia, hypoglycemia, poor peripheral perfusion and other complications related to poor neonatal transport was another challenge for survival and good outcomes of referral neonates**.** The death occurred about 21.2–79.1% in hypothermic and 60–75.29% in poor peripheral perfusion neonates at admission [12, 13]. The incidence of death and hypoglycemia from referred neonates with oxygen saturation < 90% at admission was 60 and 14.6% respectively. The mortality risk of clinically unstable neonates at admission time was 5 times higher than those referred in a stable condition [14–16].

In resource-limited countries referral systems is not well established and referral neonates travel long distances without emergency care, resuscitation equipment and adequately trained personnel to get access to specialty care [17–19]. Many studies have shown that long-distance travel without emergency care and inadequate continuity of care during transfer, sociodemographic factors, neonatal and maternal factors and health-related factors affect the health of neonates [20–23]. Data on the incidence and predictive factors associated with mortality among referred neonates are a crucial and timely issues. Therefore, this study aimed to assess the incidence and predictors of neonatal mortality among referred neonates admitted to comprehensive and specialized hospitals in Amhara regional state, North Ethiopia 2020.

## Method and materials

### Study setting and design

A prospective follow-up study was conducted from October 1 to November 30, 2020, in neonatal intensive care units of comprehensive specialized and specialized hospitals in the Amhara regional state including Debre Markos and Felege Hiwot comprehensive specialized hospitals and Tibebe Ghion and Gonder specialized hospitals. Felege Hiwot and Tibebe Ghion hospital are found in Bahi Dar (the capital city of Amhara regional state) 565 km far from Addis Ababa. Debre Markos and Gonder hospitals are found to be 299 and 730 km respectively far from Addis Ababa. These Hospitals are final referral choices for other health institutions around that provide tertiary level neonatal care. Hospitals have neonatal intensive care units with 158 neonatal beds organized with necessary materials and equipment and mixed health professionals (neonatal and general nurse, general practitioners, pediatricians, and other staff). The major services include general neonatal care services, blood and exchange transfusion, phototherapy, and ventilation support such as continuous positive air pressure (CPAP).

Hospitals had an average of 454 two-months neonatal admissions referred from other health institutions around hospitals: Debre Markos, Felege Hiwot, Tibebe Ghion, and Gonder comprehensive specialized hospitals have an average of 86, 121, 106, and 141 referrals neonatal admission respectively. The Amhara regional state has only six comprehensive and two specialized hospitals in 2020. These hospitals may have many referred neonates. Geographically hospitals are far from many primary and secondary health centers and take a long time to transport referred neonates from catchment areas. That is why this setting was chosen as the study area.

### Populations

All neonates referred to the comprehensive and specialized hospitals in the Amhara regional state from other health institutions in 2020 were the source population.

All referred neonates who were referred and transferred to study areas of hospitals and admitted in the neonatal intensive and emergency care units during the study period were taken as the study population.

### Eligibility criteria

All inter referral neonates from other health facilities and admitted to the neonatal intensive and emergency care units were included.

All self-referral neonates who came by their preference without health professional decisions and all neonates readmitted after discharge with improvement were excluded.

### Sample size determination

The Sample size was determined by the Fleiss formula using Epi Info7.2.1 by considering significant predicting variables from related works of literature (Table 1). Thus, sample size calculation was based on the following assumptions (Two-sided confidence level = 95%, power = 80% and ratio of exposure to non-exposure = 1:1) and from a prospective follow up study conducted on predictors of mortality in referred neonates with neonatal sepsis at a tertiary care center Maharashtra, India the proportion of neonatal death from hypothermic as exposed and non-hypothermic as non-exposed and odds ratio with non-response rate 10% [12].

$$ n1=\frac{\ {\left(Z\frac{\alpha }{2}+Z1-\beta \right)}^2 pq\left(r+1\right)}{r{\left(P1-q1\right)}^2} $$

**Table 1:** sample size calculation to assess the incidence and predictors of neonatal mortality among referred neonates admitted to Comprehensive Specialized Hospitals in Amhara Regional State North, Ethiopia, 2020

Significant factors associated with neonatal mortality	Proportion among exposure and non-exposure	Total sample size	Source
Capillary refill time:			
> 3 s	P1 = 0.45	238	(16)
< 3 s	q1 = 0.26		
Body temperature at admission time:			
Hypothermic	P1 = 0.43	396	
Non-hypothermic	q1 = 0.29		
Delivery conducted:			
Unskilled birth attendant	P1 = 0.73	42	(48)
Skilled birth attendants	q1 = 0.26		
Oxygen saturation < 90%	P1 = 0.36	92	(24)
Oxygen saturation > 90%	q1 = 0.11		

Where, n1 is the sample size.

p1 is the proportion of death among exposed.

q1 is the proportion of death among unexposed.

r ratio of unexposed to exposed.

p proportion of death from the total number of populations in the sample.

q = 1-p.

Zα/2 is standard normal deviation for two-tailed tests based on alpha level (relates to confidence interval level) and Z1-β is standard normal deviation for a one-tailed test based on beta level. Thus, by adding 10% non-response rate the final sample size was 436.

### Sampling technique

All referred neonates admitted in NICUs in each hospital between October 1 to November 30, 2020, that meet the inclusion criteria and consented willingly to participate in the study were recruited. First, the total sample was proportionally allocated for each hospital and then individuals were entered into the study consecutively. The average referred neonatal admission in the previous 2 months in four hospitals was 454: Debre Markos, Felege Hiwot, Tibebe Ghion and Gonder Hospitals have an average of 86, 121, 106, and 141 referred neonatal admission respectively. Eighteen neonates were excluded from the study due to exclusion criteria. The selected participants were followed a maximum of 28 postnatal days from admission till the outcome of interest. The study was based on primary and secondary data.

### Operational definitions

**Referred neonates**: are all sick neonates referred and transferred to study area hospitals from other health institutions for special care.

**Incidence of referred neonatal mortality**: is the probability of neonatal death referred from other health facilities from admission to the 28th day of birth.

**Duration of transport:** average time spent measured in minutes to transport neonate from last 11 referring health facilities to study hospitals as reported by the respondent.

**Referral points**: number of referral chain the neonate have before reaching Hospital.

**Single referral point**: having only one referral point from other health facilities.

**Multiple referral**s: two or more referral points from other health facilities.

**Vital sign monitoring:** is measuring and checking any vital signs during transportation of referred neonate.

**Intra transport resuscitation:** is any treatment given such as oxygen administration, fluid resuscitation, temperature monitoring, and breastfeed during transportation of referred neonate.

### Data collection tool and procedure

Data collection tools were adapted from related works of literature and guidelines [21, 23–26] and prepared in the English language for the pretest. Data were collected by a semi-structured questionnaire and checklist for observation and chart review. Both primary and secondary data were used. The prepared tool comprised of socio-demographic, maternal and health, neonatal and referral factors.

Before the study, the period begins an adequate number of data collectors working in the NICU were assigned and taken one-day training. The questionnaires were pretested with 10% of participants before the actual data collection period to see consistency in the recording of variables and clarity of questions. Data were collected by eight nurses face to face interviewing of caregivers, observing and reviewing patient’s medical chart using semi-structured questioner with continuous supervision.

During admission, the current physiologic status of the neonate was evaluated and all necessary information was taken and followed from admission to 28 post-natal days. In the end, participants were classified as not died if the participant is improved and discharged, lost to follow up if communication ended before follow-up period and died if he/she died in hospital before completing follow up period [27]. The outcome was recorded as survived or died.

### Data quality control

To achieve data quality the data collection tool was prepared by reviewing related works of literature. Before actual data collection, a pretest was done with 10% of participants at Finote Selam secondary hospital and the appropriateness of the data collection questionnaire and necessary modifications were made on the consistency of the recording of variables and clarity of questions. Staff nurses who were working in NICUs and preferably who had taken basic NICU training were involved in the data collection and one supervisor in each hospital was assigned. One-day training was given for both data collectors and supervisors concerning the data collection tool and data collection process. The supervisors were followed closely and supervise throughout the entire data collection period. The consistency in the recording of variables during follow-up was checked by taking a few patients and amendments were done on the data collection tool.

### Data processing and analysis

Data were entered into Epi-data™ Version 4.2 after checking the completeness and consistency and then exported into STATA™ Version 14 for data recording, cleaning and analysis. Then after the outcome of each study participant was dichotomized into died or survived. A bi-variable logistic regression model was fitted for each explanatory variable. Moreover, those variables having a *p*-value < 0.25 in the Bivariable logistic regression model were selected for multivarible analysis. The odds ratio with its 95% confidence interval and *p*-values was calculated. In the multivariable logistic regression analysis explanatory variables with *p*-values < 0.05 were considered as statistically significant and predictors of mortality. Multicollinearity was checked by a variance inflation factor. Hosmer-Lemeshow goodness of fit test was conducted to ascertain whether the model was correctly specified or data conflicted with assumption was made by the model. The result was presented using tables, graphs, figures and text.

## Results

### Sociodemographic factors

In this study, there was 436 neonatal admission in NICUs of Debre Markose, Felege Hiwot, Tibebe Ghion and Gonder specialized hospitals. The analysis was done on a total of 422 patients. The response rate of this study was 100% with 216 (51.19%) of neonates being males and two hundred thirty-six (55.92%) from the rural area. The Median neonatal age at admission was 2 days with IQR 2–4 days. Two hundred fifty-nine (61.37%) neonates were admitted to NICUS within 2–7 days after birth. The mean (±SD) age of the mothers was 28 (±3.2) years. Two hundred nineteen (51.90%) mothers were 25–34 years age range. One hundred thirty-three (31.51%) neonates were born from mothers who can not read and write and 244(57.82%) mothers are housewives (Table 2).

**Table 2:** Sociodemographic characteristics of referred neonates in Amhara regional state comprehensive specialized and specialized Hospitals from October 1 to December 30, 2020 (*N* = 422)

Variables	Categories	Frequency	Percent (%)
Sex of neonate	Female	206	48.81
	Male	216	51.19
Age neonate (days)	⦤ 1 day	124	29.38
	2–7 days	259	61.37
	8–28 days	39	7.35
Age of mother (years)	15–19	31	7.35
	20–24	91	21.56
	25–34	219	51.90
	≥35	81	19.19
Residence	Urban	186	44.08
	Rural	236	55.92
Maternal occupation	Housewife	244	57.82
	Employee (GO and NGO)	72	17.06
	Private work	106	25.12
Maternal education	Can’t read and write	133	31.51
	Primary school	104	24.64
	Secondary school	65	15.40
	Higher education	120	28.45

*GO* Government organization, *NGO* Non-Governmental Organization

### Maternal and Health service-related factors

Almost half (48.34%) of neonates were delivered from mothers who completed four and more ANC visits. Two hundred forty-five (58.06%) and 259 (61.37%) neonates were delivered from multigravida and para (2–4) mothers respectively. Three hundred fourteen (73.41%) neonates were born in a health facility and attended by health personnel. Three hundred ten (73.46%) neonates are vaginal delivery (Table 3).

**Table 3:** Maternal and health service factors of referred neonates in Amhara regional state comprehensive specialized and specialized Hospitals from October 1 to December 30, 2020 (*N* = 422)

Variables	Categories	Frequency	Percent (%)
ANC follow up	0	25	5.92
	1	14	3.32
	2–3	179	42.42
	⦥4	204	48.34
Gravidity	1	117	27.73
	2–4	245	58.06
	⦥5	60	14.23
Parity	1	117	27.73
	2–4	259	61.37
	⦥5	46	10.90
Place of delivery	Home	108	25.59
	Health facility	314	73.41
Delivery mode	Spontaneous vaginal delivery	310	73.46
	Cesarean section	42	9.95
	Device assisted	70	16.59
Birth attendants	Traditional	108	25.59
	Skilled	314	74.41
APH	Yes	18	4.27
	No	404	95.73
PIH	Yes	11	2.61
	No	411	97.39
HIV status	Positive	7	1.66
	Negative	405	95.97
	Unknown	10	2.37

*APH* Antepartum Hemorrhage, *PIH* Pregnancy Induced Hemorrhage, *HIV* Human Immune Virus

### Neonatal related factors

The mean (±SD) admission weight of neonates was 2333.18 (±312.5) gram. One hundred seventy-five (41.47%) neonates were admission weight 1500–2500 g range. During admission two hundred ninety-six (70.14%) neonates were hypothermic, one hundred ninety-eight (46.92%) had hypoglycemia and two hundred twenty-eight (54.03%) were peripheral oxygen saturation less than 90% measured at admission time. Two hundred-sixty four (62.55%) neonates were born after 37 weeks of pregnancy (Table 4).

**Table 4:** Neonatal related factors of referred neonates in Amhara regional state comprehensive specialized and specialized Hospitals from October 1 to December 30, 2020 (*N* = 422)

Variables	Categories	Frequency	Percent
Admission weight (gm)	1000–1500	74	17.53
	1500–2500	175	41.47
	≥2500	173	41.00
CRT less than 3 s at admission	Yes	45	10.66
	No	377	89.34
Hypothermia at admission	Yes	296	70.14
	No	126	29.86
Types of pregnancy	Single	353	83.65
	Twin	69	16.35
Oxygen saturation at admission	Yes	194	45.97
	No	228	54.03
Gestational age	< 32 weeks	55	13.03
	32–36 Weeks	103	24.40
	≥37 weeks	264	62.55
Hypoglycemia at admission	Yes	198	46.92
	No	224	53.08

*CRT* Capillary Refill Time

### Referral related factors

Three hundred eleven (73.70%) had been referred by ambulance. Three hundred twenty-six (77.25%) were followed by health personnel and 317(75.12%) had no intra-transport resuscitation. Two hundred twenty-five (53.32%) were vital signs checked during transfer. The mean distance travel was 107.77 with a range of 44.53–171.01 min. One hundred seventy-two (40.76%) neonates traveled ≥120 mins to access specialty care. About 25.83% of participants cross two or more referral points (Table 5).

**Table 5:** Referral-related factors of referred neonates in Amhara regional state comprehensive specialized and specialized Hospitals from October 1 to December 30, 2020 (*N* = 422)

Referral related factors	Response /Categories	Frequency	Percent (%)
Vital sign monitoring on transportation	Yes	225	53.32
	No	197	46.68
Referral notes	Yes	387	91.71
	No	35	8.29
Referred by Ambulance	yes	311	73.70
	No	111	26.30
Intra transport resuscitation	Yes	105	24.88
	No	317	75.12
Referral point	One	313	74.17
	Two or more	109	25.83
Health personnel with referred neonate	Yes	326	77.25
	No	96	22.75
Travel time (minutes)	≤30	61	14.45
	31–60	64	15.17
	61–120	125	29.62
	≥120	172	40.76
Resuscitation equipment (Ambubag and face mask)	Yes	112	26.54
	No	310	73.46

**Fig. 1:**
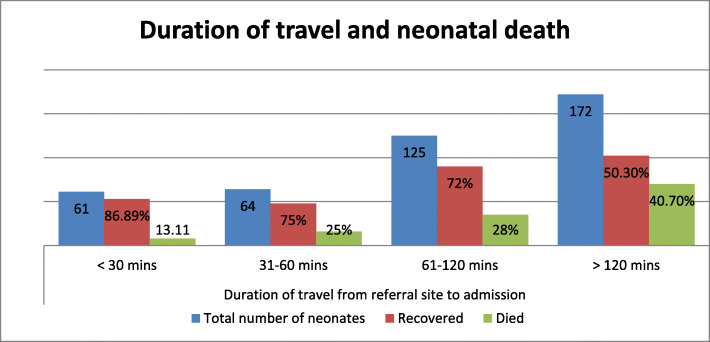
Shows duration of travel and referred neonatal deaths in each category in Amhara regional state comprehensive specialized and specialized Hospitals from Oct 1 to Dec 30, 2020 (*N* = 422)

From the total of referred neonates 172 neonates travel > 120 mins of distance and 40.70% of neonates died. The graph shows referral neonates who travel long distances have a high probability of death than those who travel short distances (Fig. 1).

### Incidence of referred neonatal mortality

This result showed that the overall mortality rate was found to be 30.60 deaths per 100 referred neonates [95% CI: 26.34–35.16]. The incidence proportion of death respective to completed days was 34.68, 30.88, and 15.38% among neonates referred within 1, 2–7, and 8–28 completed days respectively. From total death 60 (46.51%) were males. About 23 (17.83%) deaths were due to sepsis, 32 (24.80%) premature, 40 (31%) perinatal asphyxia, 3(2.33%) congenital malformation and 31(24.03%) deaths were due to other causes.

### **Predictors** of neonatal mortality

In the Bi-variable logistic regression analysis, variables having a *p*-value < 0.25 were fitted into the multivariable logistic regression analysis. Likewise, in the bi-variable analysis, place of delivery, mode of delivery, types of pregnancy, gestational age, hypothermia, hypoglycemia, oxygen saturation at admission, prolonged CRT, admission weight, distance traveled to reach referral hospital, age of neonate at admission, vital sign monitoring, mode of transportation, and residence were found to be a candidate for the multivariable logistic regression analysis.

Finally, in the multivariable logistic regression analysis, hypothermia, hypoglycemia and oxygen saturation < 90% at admission, distance to reach referral hospitals, admission weight, home delivery, and age of neonate at admission were found significant predictor of neonatal death among referred neonates who were admitted at NICUs of Amhara regional state comprehensive specialized and specialized hospitals.

This study showed referral neonates who were delivered at home by traditional birth attendants were 2.8 [AOR = 2.89, 95% CI:(1.64–4.77)] times higher risk of death than those who were delivered in the health facility by health professionals. Similarly, neonates who had admission weight less than 1500 g were 3.2 [AOR =3.2, 95% CI: (1.68–6.09)] times higher risk of death than those admission weights greater than 2500 g.

The present study also revealed that the odds of risk of death was 3.8 [AOR = 3.8, 95% CI: (1.65–9.14) times among those who were traveled more than 120 min to reach referral hospitals compared to neonates who travel < 30 min. Being hypothermic at admission time increases the risk of death by 2.7 times compared to non-hypothermic referred neonates [AOR = 2.7, 95%: CI (1.44–5.13).

Moreover, those neonates with oxygen saturation < 90% (SPO2) at admission time were a 1.9 [AOR = 1.9, 95% CI: (1.19–3.21)] times higher risk of death than counterparts. Hypoglycemia increases the risk of death by 1.8 times in hypoglycemic referred neonates compared to non-hypoglycemic referred neonates [AOR = 1.8, 95% CI: (1.11–3.00)].

The age of the neonate at admission time was also another significant predictor. Those neonates who referred before celebrating 1 day of birth were 3.4 [(AOR = 3.4, 95% CI: (1.23–9.84)] times at high risk of death than neonates who were referred after completing 7 days of birth (Table 6).

**Table 6:** Bi-variable and multivariable logistic regression analysis to identify the predictors of neonatal mortality among referred neonates admitted in Amhara regional state comprehensive specialized and specialized Hospitals October 1 to December 30, 2020 (*N* = 422), North, Ethiopia

Variables	Categories	Outcome status		COR (95% CI)	AOR (95% CI)
		Death	Recovered		
Place of delivery	Home	49	56	2.5(1.63–4.1)	2.8(1.64–4.77)*
	Health facility	80	237	1	1
Mode of delivery	SVD	102	208	2.08(0.93–4.67)	1.17(0.46–2.97)
	DA	19	51	1.6(0.62–4.02)	1. 18(0.40–3.45)
	C/S	8	34	1	
Types of pregnancy	Twin	33	36	2.4(1.44–4.16)	1.24(0.60–2.53)
	Single	96	257	1	1
Gestational age	<32wks	27	28	3.14(1.66–5.72)	1.76(0.78–3.96)
	32–36 weeks	39	64	1.48(1.27–3.26)	1.67(0.88–3.16)
	≥37 weeks	62	202	1	1
Hypothermia at admission	Yes	111	185	3.6(2.07–6.25)	2.7(1.44–5.13) *
	No	18	108	1	1
Hypoglycemia at admission	Yes	79	119	2.3(1.51–3.53)	1.8(1.11–3.00) *
	No	50	174	1	1
Oxygen desaturation at admission	Yes	78	116	2.3(1.52–3.56)	1.9(1.19–3.21) *
	No	51	177	1	1
Prolonged CRT	Yes	26	19	3.6(1.93–6.85)	1.86(0.85–4.07)
	No	103	274	1	1
Admission weight	< 1500 g	40	34	2.8(1.60–4.93)	3.2(1.68–6.09) *
	1500-2500 g	38	137	0.7(.40–1.078)	0.91(.62–1.92)
	≥2500 g	51	122	1	1
Distance travelled	**≥**120mins	70	102	3.5(1.66–7.36)	3.8(1.65–9.14) *
	60–120 min	33	92	1.8 (0.83–4.01)	2.15(0.85–5.41)
	30–60 min	16	48	1.7 (0.70–4.11)	1.54(0.55–4.32)
	< 30 mins	10	51	1	1
Age of neonate at admission	< 2 day	43	81	2.9(1.13–7.51)	3.4(1.23–9.84)*
	2–7 days	80	179	2.4(1.95–6.10)	2.3(0.96–5.71)
	8–28 days	6	33	1	1
Vital sign monitoring in transportation	No	67	130	1.4(0.89–2.05)	1.45(0.86–2.44)
	Yes	62	163	1	1
The mode of transportation is an ambulance	No	44	67	1.7(1.11–2.75)	1.58(0.75–4.50)
	Yes	85	226	1	1
Residence	Rural	82	154	1.5(0.98–2.29)	1.01(0.65–1.86)
	Urban	47	139	1	

*significant at*p* **< 0.05**

*SVD* Spontaneous Vaginal Delivery, *C/S* Cesearen Section, *DA* Device Assisted

## Discussion

The proportion of death among referred neonates admitted in comprehensive specialized and specialized hospitals neonatal intensive care unit in Amhara regional state was [(30.60%) 95% CI: 26.34–35.16)]. The incidence proportion of death was 34.68, 30.88, and 15.38% among neonates referred within 1, 2–7, and 8–28 days respectively. These findings were in line with studies conducted in Gondar comprehensive specialized hospital 28.8% [25], Mauritania 34.7% [28], and tertiary care teaching government hospitals in India 31.98 and 32.9% [8, 29]. However, study finding was higher than previous studies conducted in Ethiopia; Debre Markos referral hospital 21% [22], Amhara regional state referral hospitals 18.6% [21], Tigray 6.04% [30], referral hospital in southern Ethiopia [31] and Nekemte Referral Hospital 8.8% [32] in southern India; 22.8% [33], 20.75% [24], 18.36% [23] and Argentina 17.5% [34]. The higher incidence of death in the present study may be due to a lack of a well-established mobile neonatal intensive care unit with adequately trained manpower that replaces care in NICU of referral Hospitals. For some studies, this might be due to the difference in the study setting which is only referral admissions; vulnerable group, study period, and geographical area.

In contrast, the study finding was lower than previous studies conducted in Guinea 46.8% [19], University Hospital of the West Indies in Jamaica 36% [35], and Bangladesh 54.5% [36]. The discrepancy might be due to the study period and sample size for some studies and other study population was focused on only neonates who were most vulnerable groups [19].

In this study, home delivery was found to be an important predictor of neonatal mortality for those neonates referred to higher specialty care. This finding is in agreement with other previous studies conducted in Gondar Comprehensive Specialized Hospital [25], Bangladesh [36], and India [23]. The possible explanation could be related to a low level of care received at home without any skilled attendants during labor and immediately after birth [26, 37]. Seventy percent of newborns delivered at home in this study were passed two or more referrals, which made delay to receive life-saving interventions.

The present study also showed that the time taken to reach referral hospital > 2 h increases mortality significantly and was found to be an independent predictor for neonatal death. This finding was consistent with the previous studies conducted in Ethiopia [37], Ghana [38], Nigeria [39], and India [8, 13, 23, 40]. Distance to specialty care is an important risk factor for early neonatal mortality. Proximity to health services and a higher level of care associated with lower early neonatal mortality [26, 37].

Also, the present study shows neonates who had hypothermia and oxygen saturation < 90% during admission were at higher risk of death than those who had no hypothermia and oxygen saturation > 90%. Hypothermia and oxygen saturation < 90% during transportation was an independent predictor of neonatal death. This study is similar to a previous study conducted in Ethiopia [11], Mauritania [28], and India [2, 12]. Lack of emergency care on the way to higher centers, poor stabilization before referral and inadequate care during transport increase clinical instability [2, 41].

This study also found hypoglycemia as predicting factor for referred neonatal death. Neonates who have hypoglycemia at admission was 1.8 time at high risk of death than those who were not hypoglycemic. The study conducted in Ethiopia [25], India [40, 42] supports the present study. This may be due to long-distance travel without feeding babies during transportation (If feeding is not contraindicated for some disease pathology), especially for those > 120 mins of distance. Moreover, admission weight less than 1500 g was a predictor of referred neonatal mortality. This finding was similar to studies in India [2, 12] and Guinea [19]. This finding is supported by the clinical practice that very low birth weight neonates are highly affected and lead to death due to vulnerability to the occurrence of life-threatening complications like hypothermia, hypoglycemia, and risk for Hospital-acquired infections.

In this study, the age of neonates was also predicting factor for neonatal death. Neonates referred within 1 day after birth was 3.4 times at high risk than neonates who referred after 7 days of birth. This study was contradicted studies in India [12, 23]. The possible reason my in clinical practice the first 24 h are life-threatening period, referring neonate without pre-referral stabilization and well-established mobile NICU increase risk of death.

## Conclusions

In general, the overall magnitude of referred neonatal mortality was 30.57% which was high. Referring without continuity of care similar to care in the NICU lead to a risk of aggravation of the newborn’s condition. The acute neonatal physiology is affected during the transport and adversely affects the outcome. Vital sign monitoring and early recognition of acute physiology of newborns and managing complications, help recovery and decrease mortality. Admission weight, hypothermia, hypoglycemia, travel distance 120 and greater than 120 min, oxygen saturation, age of neonate at admission, and home delivery were independent predictors of mortality. Referring neonates by ambulance accompanied by skilled personnel and emergency resuscitation equipment through close communication and establish a system or referral network that would facilitate transfers to reduce travel and waiting time was recommended. Information pertaining to the time of hospitalization, baby’s temperature and breastfeeding status during transportation which is pertinent to know the hypothermia and hypoglycemia were not addressed in this study.

## Data Availability

On reasonable requests, the full data set and other materials related to this study can be obtained from the corresponding author.

## Abbreviations

ANC: Antenatal Care
CBN: Community Based Nutrition
CBNC: Community based New Born Care
CEOMNC: Comprehensive Emergency Obstetric Management Newborn Care
CPAP: Continuous Positive Air Pressure
CRT: Capillary Refill Time
CSH: Comprehensive Specialized Hospital
DMCSH: Debre Markose Comprehensive Specialized Hospital
EDHS: Ethiopian Demographic Health Survey
FHCSH: Felege Hiwot Comprehensive Specialized Hospital
GCSH: Gonder Comprehensive Specialized Hospital
NETS: Neonatal Emergency Transport Service
KMC: Kangaroo Mother Care
LAMA: Left Against Medical Advice
MDG: Millennium Development Goal
NETSs: Newborn Emergency Transport Services
NGOs: Non-Governmental Organization
NICU: Neonatal Intensive Care Unit
NMR: Neonatal Mortality Rate
PNC: Post Natal Care
SDG: Sustainable Development Goal
SSA: Sub-Saharan Africa
SVD: Spontaneous Vaginal Delivery
TGCSH: Tibebe Gion Comprehensive Specialized Hospital
TRIPS: Transport Risk Index of Physiologic Stability
TOPS: Temperature, Oxygen saturation, Perfusion and Blood sugar
UNICEF: United Nations International Children’s Emergency Fund
